# Hypovitaminosis D in patients undergoing kidney transplant: the importance of sunlight exposure

**DOI:** 10.6061/clinics/2017(07)05

**Published:** 2017-07

**Authors:** Cristiane F. Vilarta, Marianna D. Unger, Luciene M. dos Reis, Wagner V. Dominguez, Elias David-Neto, Rosa M. Moysés, Silvia Titan, Melani R. Custodio, Mariel J. Hernandez, Vanda Jorgetti

**Affiliations:** I Divisao de Nefrologia, Faculdade de Medicina FMUSP, Universidade de Sao Paulo, Sao Paulo, SP, BR; II Divisao de Urologia, Faculdade de Medicina FMUSP, Universidade de Sao Paulo, Sao Paulo, SP, BR; III Mestrado em Medicina, Universidade Nove de Julho (UNINOVE), Sao Paulo, SP, BR; IV Servicio de Nefrología y Trasplante Renal, Hospital Universitario de Caracas, Universidad Central de Venezuela, Caracas, Venezuela

**Keywords:** Vitamin D, Kidney Transplantation, Parathyroid Hormone, Hypovitaminosis D, Chronic Kidney Disease

## Abstract

**OBJECTIVES:**

Recent studies have shown a high prevalence of hypovitaminosis D, defined as a serum 25-hydroxyvitamin D level less than 30 ng/ml, in both healthy populations and patients with chronic kidney disease. Patients undergoing kidney transplant are at an increased risk of skin cancer and are advised to avoid sunlight exposure. Therefore, these patients might share two major risk factors for hypovitaminosis D: chronic kidney disease and low sunlight exposure. This paper describes the prevalence and clinical characteristics of hypovitaminosis D among patients undergoing kidney transplant.

**METHODS:**

We evaluated 25-hydroxyvitamin D serum levels in a representative sample of patients undergoing kidney transplant. We sought to determine the prevalence of hypovitaminosis D, compare these patients with a control group, and identify factors associated with hypovitaminosis D (e.g., sunlight exposure and dietary habits).

**RESULTS:**

Hypovitaminosis D was found in 79% of patients undergoing kidney transplant, and the major associated factor was low sunlight exposure. These patients had higher creatinine and intact parathyroid hormone serum levels, with 25-hydroxyvitamin D being inversely correlated with intact parathyroid hormone serum levels. Compared with the control group, patients undergoing kidney transplant presented a higher prevalence of 25-hydroxyvitamin D deficiency and lower serum calcium, phosphate and albumin but higher creatinine and intact parathyroid hormone levels.

**CONCLUSIONS:**

Our results confirmed the high prevalence of hypovitaminosis D in patients undergoing kidney transplant. Therapeutic strategies such as moderate sunlight exposure and vitamin D supplementation should be seriously considered for this population.

## INTRODUCTION

Several studies have demonstrated a high prevalence of hypovitaminosis D (hypo D) in different regions of the world including Latin America, even though these places receive the necessary ultraviolet radiation (UVR) required to produce adequate levels of 25-hydroxyvitamin D [25(OH)D] 1-3.

Recently, vitamin D and its metabolites have been extensively studied because of the role they play in functions such as the regulation of calcium (Ca) and phosphorus (P) levels, parathyroid hormone suppression and bone mineralization in adults 4. In addition, there are associations between vitamin D deficit and the development of cardiovascular and immunological disease, cancer, diabetes mellitus and arterial hypertension 2.

Vitamin D deficiency in patients with chronic kidney disease (CKD) can lead to alterations in bone mineral metabolism, resulting in frequent fractures and increased cardiovascular mortality 4,5. In this context, increases in the serum levels of 25(OH)D at 10 ng/ml are associated with a 14% decreased mortality risk in this population 5.

Kidney transplant (KT) resolves some of these disorders and leads to improvements in the prognosis and quality of life of patients with end-stage renal disease. However, studies have also shown that hypo D is a persistent event in during both early and late transplant periods, requiring supplementation in most cases 4-10. The major causes of hypo D in patients undergoing KT include: the increased catabolism of vitamin D secondary to the use of glucocorticoids 11, restricted sunlight exposure, and sunscreen use by medical indication because immunosuppression is associated with skin cancer in these patients 12.

In addition to the above known effects, Obi et al. showed that vitamin D deficiency/insufficiency in patients undergoing KT is associated with an increased risk of renal interstitial fibrosis, renal tubular atrophy, and glomerular filtration rate (GFR) decline 13. Given the recent developments of its molecular properties, vitamin D plays a multifaceted role and has led us to determine the prevalence and clinical characteristics of hypo D in a cohort of patients with KT compared with a control group.

## PATIENTS AND METHODS

Patients undergoing KT were followed at the Transplant Unit of Hospital das Clinicas da Faculdade de Medicina da Universidade de São Paulo. Of these patients, we selected those who were older than 18 years, showed stable GFR, and were more than 1 year removed from KT. A total of 752 patients was selected (355 females). In this cohort, we determined a representative sample size calculation. In this calculation, we considered a 32.5% prevalence of 25(OH)D deficiency and 5% alpha and a 1% beta risks for a test power ratio of 99%. The final KT population was composed of 149 patients; race, gender, age, donor type, serum creatinine, proteinuria, glutamic-oxaloacetic transaminase (GOT), glutamic-pyruvic transaminase (GPT), gamma-glutamyltransferase (GGT) levels, immunosuppressive therapy, body mass index (BMI) and vitamin supplementation were controlled.

**Figure 1:**
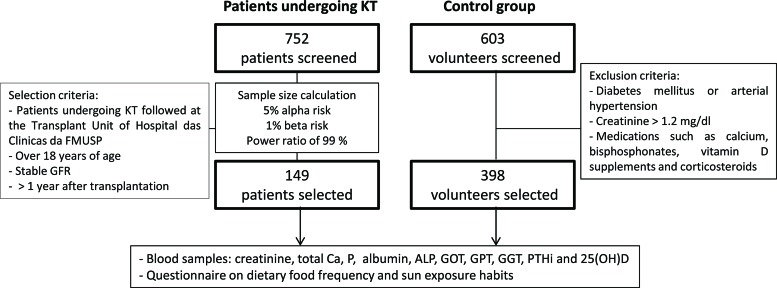
Participant data. KT: kidney transplant; GFR: glomerular filtration rate; 25(OH)D: 25 hydroxyvitamin D; Ca: calcium; P: phosphorus; PTHi: intact parathyroid hormone; ALP: alkaline phosphatase; GOT: glutamic-oxaloacetic transaminase; GPT: glutamic-pyruvic transaminase; GGT: gamma-glutamyltransferase.

Concomitantly, we selected 398 individuals (333 females) from a sample of 603 volunteering outpatient employees of the Hospital Universitário da Universidade de São Paulo and graduate students from the Faculdade de Medicina da Universidade de São Paulo previously included in Unger et al. We excluded individuals with diabetes mellitus, arterial hypertension, serum creatinine levels higher than 1.2 mg/dL or those on medications/supplements such as Ca, bisphosphonates, vitamin D and corticosteroids (see Figure 1) 2.

A blood sample was collected during late winter of 2006, and serum Ca, P, creatinine, albumin, intact parathyroid hormone (PTHi), total alkaline phosphatase (ALP), 25(OH)D and BMI were analyzed.

KT recipients were submitted to a single blood collection during late winter. We chose late winter to coincide with the period in which samples were collected from the control group and because it has less UV radiation, enabling us to detect a greater number of patients with hypo D.

The following parameters were analyzed in the selected patients: age, gender, race (white or non-white), donor type, time after KT, immunosuppressive therapy, and post-KT fractures (spontaneous or minimal trauma). In addition, the serum concentrations of creatinine, total Ca, P, albumin, ALP, GOT, GPT, GGT, PTHi and 25(OH)D were measured. The GFR was estimated using the MDRD simplified formula 14.

The patients completed a questionnaire on dietary food frequency that evaluated the consumption frequency of major food sources of vitamin D (e.g., fatty fish and fish oil). Sunlight exposure habits were analyzed based on the frequency of exposure (3 or more times a week or less than 3 times a week) and a sunscreen protection factor (yes or no).

Total serum Ca, serum P, albumin, ALP, GOT and GGT were determined using routine laboratory techniques. PTHi was measured using the Immulite analyzer (Diagnostic Products Corporation, Los Angeles, CA, USA; reference range, 10-87 pg/ml). 25(OH)D was measured using a chemiluminescent assay (Dia-Sorin Inc., Stillwater, MN, USA). Serum concentrations of 25(OH)D between 15 and 30 ng/mL were considered insufficient, and concentrations less than 15 ng/ml were considered deficient.

All procedures in this study were performed after approval from the local institutional committee, Comissão de Ética para Análise de Projetos de Pesquisa (CAPPesq n° 1035/07), and in accordance with the ethical standards of the 1964 Helsinki Declaration.

### Statistical analysis

The data are expressed as either means ± SD or medians and ranges; categorical data are described as percentages.

To determine the relationship between the 25(OH)D levels and other categorical variables, we used chi-square test and, when necessary, Fisher’s exact test.

We used the Box and Cox transformation for the variables age, time after KT, albumin, creatinine, PTHi, GOT, GPT, GGT and ALP to ensure the normality assumption. Even with the Box and Cox transformation, the variables age, time after KT and GOT did not reach normality. Because these transformed variables presented homoscedasticity (according to Levene’s test), they were also considered normally distributed to compare the serum 25(OH)D levels.

An ANOVA was used to determine the relationship between the clinical and biochemical variables and serum 25(OH)D (deficient, insufficient or normal). If results were significant, then we applied the Bonferroni post hoc test. The correlation between the 25(OH)D levels and other biochemical parameters was analyzed using Spearman’s correlation coefficients.

The patients undergoing KT and the control group differed with regard to gender; therefore, we used an ANOVA adjusted for gender to compare these two groups with regard to BMI, P, Ca, albumin, creatinine, PTHi, ALP and 25(OH)D for each variable separately.

The level of significance was set at 5% (*p*-value<0.05).

## RESULTS

### Basic characteristics of the patients undergoing KT

Of the 149 patients undergoing KT, 56.4% were female, and 68.4% were white. The median age was 44 (18 - 81) years, and the median time after KT of was 6 (1 - 36) years. Approximately 55% of these patients received a graft from a living donor.

Regarding their 25(OH)D serum levels, 56 (37.5%) patients presented deficiency, 62 (41.6%) insufficiency, and only 31 (20.8%) a normal concentration. Thus, the prevalence of hypo D in the study sample was 79.2%.

Post-KT insufficiency fractures (spontaneous or minimal trauma) were reported in 10% of the patients (N=15). The mean 25(OH)D serum level was similar between patients without and those with fractures (21.8±13.2 ng/mL *vs*. 21.9±8.3 ng/mL). Likewise, we found no differences between patients with and without fractures regarding the rates of 25(OH)D deficiency or insufficiency.

In addition to the data described above, Table 1 shows the demographic and biochemical parameters. The patients undergoing KT showed serum PTHi above the reference range. We did not find differences in the 25(OH)D levels (21.1±12.1 *vs*. 22.6±13.4) between patients with abnormal liver function (GGT levels>50 U/L) and those with preserved liver function.

### Clinical and demographic characteristics according to the 25(OH)D categories among patients undergoing KT

Table 2 describes the clinical and demographic characteristics according to the 25(OH)D categories (deficient, insufficient and normal). Donor type was associated with important differences, although they were not significant. Specifically, we found that 28% of patients who received a graft from a living related donor had normal 25(OH)D levels, whereas only 12% of patients receiving a graft from a standard cadaveric donor showed normal 25(OH)D levels (*p*=0.055).

Regarding the frequency of sunlight exposure, we observed a significant difference between those who were exposed to the sun more than 3 times a week and those who had less exposure with regard to 25(OH)D levels; specifically, 55.8% of patients with higher sun exposure had a normal concentration of 25(OH)D *versus* 10.4% in the group with lower sun exposure (*p*<0.0001). The use of sunscreen did not affect the serum concentrations of this vitamin. We did not find a significant relationship between 25(OH)D serum levels and consumption of food sources of vitamin D. The data are shown in Table 3.

### Biochemical parameters by 25(OH)D category among patients undergoing KT

Table 4 compares the biochemical parameters across 25(OH)D categories. The results revealed that only serum creatinine and PTHi significantly differed with regard to 25(OH)D levels. Specifically, we found lower serum levels of creatinine and PTHi among patients with normal 25(OH)D.

### Comparison of the demographic and biochemical parameters between the control group and patients undergoing KT

Table 5 shows that the control group and patients undergoing KT had different distributions based on gender. Regarding the biochemical data, we found significant differences in creatinine, albumin, Ca, P and PTHi between men and women. As expected, patients undergoing KT showed higher levels of creatinine and PTHi as well as lower levels of P than the control group.

However, patients undergoing KT presented lower levels of this hormone, regardless of 25(OH)D category, when compared to the control group (*p*=0.001); these data are shown in Table 6.

We analyzed the correlations between the serum 25(OH)D levels and biochemical parameters but found only one negative correlation with regard to serum PTHi levels (r=-0.24; *p*<0.03).

## DISCUSSION

Despite the growing interest in vitamin D status and its supplementation forms in both the normal population and patients with CKD, a lack of data remains regarding vitamin D levels in KT recipients. Little research has compared the 25(OH)D serum levels between patients undergoing KT and the healthy population. Our findings coincide with those of Ebbert et al., who analyzed a pediatric population, reporting the presence of hypo D in patients undergoing KT as well as control groups 15. However, our findings showed that patients undergoing KT had a higher prevalence of deficiency.

The current study demonstrated that 79% of patients undergoing KT at the Hospital das Clinicas da Faculdade de Medicina da Universidade de São Paulo presented with an insufficiency or deficiency of 25(OH)D during late winter. These results are similar to those obtained in other studies conducted throughout the world and across climatic seasons, revealing a total approximate prevalence of 76-97% 7-10.

Stavroulopoulos et al. analyzed patients undergoing KT during the summer and winter and confirmed that levels of 25(OH)D are lower during winter 7. That study explains why the current evaluation was performed during late winter, i.e., to detect the highest possible number of patients with hypo D. Researchers who plan future studies on vitamin D and clinicians who monitor and manage vitamin D deficiencies in patients undergoing KT must consider this seasonal variation.

Nutritional status did not affect serum 25(OH)D levels, and in contrast to other published data, we did not find a higher prevalence of hypo D in obese patients, even though the bioavailability of vitamin D can be influenced by its storage in adipocytes 16,17.

Hypo D is one of the major causes of secondary hyperparathyroidism (SHPT). Unger et al. showed that healthy subjects develop SHPT in the context of vitamin D deficiency during winter but show improvements in summer 2. Therefore, this seasonal variation in 25(OH)D and PTH is likely a normal response to physiologically compensate for the drop in vitamin D levels during winter. Among patients undergoing KT, studies have been conducted during different periods of the year, making it difficult to know whether they follow the trend observed in the general population. The literature commonly describes parathyroid hormone stimulation due to 25(OH)D deficiency during the earliest periods after KT as a consequence of the high doses of corticosteroids and persistent elevation of phosphatonins 6,18,19. Some authors have also found this correlation during later periods of KT recovery 7,20. We observed the same trends in our patients, showing a negative correlation between PTHi and 25(OH)D serum levels.

In addition, regarding the biochemical associations described above in the general population, vitamin D insufficiency contributes to the development of osteoporosis. Moreover, adequate supplementation can reduce the fracture risk in approximately 20% of this population 21. However, the effect of this hormone on the prevalence of fracture during later periods of KT is unclear, which suggests that low mineral bone density and hypo D are multifactorial conditions 7. In this regard, we found no difference between patients with and without fracture.

The reasons for hypovitaminosis after KT include low sunlight exposure and the accelerated catabolism of vitamin D secondary to glucocorticoids use 22. Exposure to UV-A and UV-B radiation via sunlight can induce skin cancer through induced mutations in the DNA structure. This radiation is clearly the most important physical environmental carcinogen for the development of skin cancer, and non-melanoma skin cancer (NMSC) is the most common malignancy among patients undergoing KT 23,24. The risk of developing this complication is associated with the duration and intensity of immunosuppression and cumulative sun exposure 18. However, some guidelines suggest that sunlight exposure for short periods of time is beneficial and does not increase the risk of skin cancer 25,26.

In this sense, sunlight exposure at least 3 times a week predicted higher 25(OH)D serum levels in our patients, despite the use of sunscreen. However, Penny et al. found an association between low 25(OH)D levels and a high incidence of NMSC, limiting the use of this therapeutic strategy to low-risk groups defined by skin type, age, gender and immunosuppressive regimen 18,27.

Regarding the effect of immunosuppression on vitamin D status after KT, steroids accelerate catabolism of vitamin D. Despite not knowing the steroid doses in our patients, the development time of transplantation suggests that they received a minimal and stable glucocorticoid dose, which most likely would not explain their vitamin D deficiency. Regarding other immunosuppressive drugs (e.g., calcineurin inhibitors), we similarly did not find an association, contrary to results reported by Filipov et al. and Eyal et al., who showed that high tacrolimus doses are associated with hypo D through hepatic CYP3A4 suppression 28,29.

The potent immunomodulatory activity of 25(OH)D through the suppression of T cell receptor proliferation and its direct effect on B cells through the inhibition of immunoglobulin production are additional physiological effects to consider among patients undergoing KT 30. Sezer et al. demonstrated that patients undergoing KT showed an association between 25(OH)D deficit and renal graft failure after one year 31, whereas other authors have shown a direct relationship between 25(OH)D deficit and renal graft survival 11,32. In this respect, our patients presented with an association between low serum levels of 25(OH)D and higher creatinine serum levels. In addition to the clinical aspects described above, Bienaimé et al. found a correlation between vitamin D status and graft histological findings; specifically, 25(OH)D serum levels 3 months after transplantation independently predictor the progression of interstitial fibrosis and tubular atrophy (IF/TA) at 12 months 32.

Other studies have shown an association between hypo D and the reception of a renal graft from a cadaver donor 32. In this regard, although our study was retrospective and did not evaluate the characteristics of the renal graft, we found a higher percentage of patients with normal 25(OH)D levels (28%) in the group receiving a renal graft from a living donor, compared to only 12% in the group receiving a renal graft from a cadaveric donor. These results suggest that the immunological tolerance in these patients could be due to the vitamin D status and that the concentration of this hormone depends on the type of renal graft received. Specifically, better compatibility conditions ensure a briefer cold ischemia time, and a minor ischemia-reperfusion effect predicts higher deposits of 25(OH)D.

In conclusion, vitamin D deficiency is prevalent among patients undergoing KT, even in geographic areas with high UV exposure. Additional studies are needed to clarify the role that donor type plays in vitamin D deficiency. Furthermore, studies are needed to define the efficacy and safety of UVR exposure as a therapeutic strategy for these patients. However, individual sunlight exposure in moderation combined with vitamin D supplementation are the easiest and safest forms to guarantee adequate levels of 25(OH)D, thereby leading to the beneficial effects of this hormone.

## AUTHOR CONTRIBUTIONS

Vilarta CF participated in study design, data collection, analysis and interpretation, and manuscript writing. Unger MD, dos Reis LM, Dominguez WV, David-Neto E, Moyses RM and Custodio MR participated in data interpretation and manuscript writing. Titan S and Hernandez MJ participated in statistical analysis, data interpretation and revision of the edited manuscript. Jorgetti V participated in study design, data analysis and interpretation, and manuscript writing.

## Figures and Tables

**Table 1:** Demographic and biochemical parameters of patients undergoing KT.

Demographic parameters		Biochemical parameters		Reference range
*Gender*		25(OH)D (ng/mL)	21.8 ± 12.8	
Female	84 (56)	*25(OH)D category*		
Male	65 (44)	Normal	31 (21)	>30 ng/ml
*Race*		Deficiency	56 (37)	<15 ng/ml
White	102 (68)	Insufficiency	62 (42)	15 - 30 ng/ml
Non-white	47 (31)	PTHi (pg/mL)	80.1 ± 78.3	10 - 87 pg/ml
Time after KT(years)	6 (1 - 36)	Ca (mg/dL)	8.9 ± 0.6	8.7 - 10.3 mg/dL
Age (years)	44 (18 - 81)	P (mg/dL)	3.0 ± 0.8	2.5 - 4.5 mg/dL
*Type of donor*		Creatinine (mg/dL)	1.5 (0.6 - 4.5)	0.5 - 1.0 mg/dL
Living related donor	82 (55)	Albumin (g/dL)	4.4 (3.4 - 5.5)	3.5 - 5.0 g/dL
Standard cadaveric donor	67 (45)	ALP (U/L)	65.8 (29.7 - 364.5)	35 - 100 U/L
Insufficiency fracture after KT	15 (10)	GOT (U/L)	23 (6 - 121)	0 - 35 U/L
BMI (kg/m^2^)	25.4 ± 5.3	GPT (U/L)	10 (3 - 81)	3 - 36 U/L
		GGT (U/L)	28 (7 - 704)	5 - 36 U/L

Values are expressed as the mean±SD, median (range) or number of patients (%); KT: kidney transplant; BMI: body mass index; 25(OH)D: 25 hydroxyvitamin D; Ca: calcium; P: phosphorus; PTHi: intact parathyroid hormone; ALP: alkaline phosphatase; GOT: glutamic-oxaloacetic transaminase; GPT: glutamic-pyruvic transaminase; GGT gamma-glutamyltransferase

**Table 2:** Demographic and clinical parameters based on 25(OH)D category among patients undergoing KT.

25(OH)D levels	*Race*		*Gender*		*BMI*		Type of donor*		*Bone fracture after KT*		*Immunosuppressive therapy*	
	White	Non-White	Female	Male	Non-obese	Obese	Living donor	Cadaveric donor	No	Yes	CNI use	CNI non-use
*Deficient*	35 (34.3)	21 (44.7)	30 (35.7)	26 (40)	29 (36.7)	27 (38.6)	28 (34.1)	28 (41.8)	52 (38.8)	4 (26.6)	44 (41.1)	12 (28.6)
*Insufficient*	43 (42.1)	19 (40.4)	35 (41.6)	27 (41.5)	34 (43)	28 (40)	31 (37.8)	31 (46.3)	53 (39.5)	9 (60)	43 (40.2)	19 (45.2)
*Normal*	24 (23.5)	7 (14.8)	19 (22.6)	12 (18.5)	16 (20.2)	15 (21.4)	23 (28)	8 (12)	29 (21.6)	2 (13.3)	20 (18.7)	11 (26.2)
Total	102 (100)	47 (100)	84 (100)	65 (100)	79 (100)	70 (100)	82 (100)	67 (100)	134 (100)	15 (100)	107 (100)	42 (100)

Values are expressed as number of patients (%); 25(OH)D: 25 hydroxyvitamin D; BMI: body mass index; KT: kidney transplant; CNI: calcineurin inhibitor.

* Chi-square test: *p*=0.055.

**Table 3:** Sunlight exposure habits and vitamin D food source consumption frequency according to 25(OH)D category in patients undergoing KT.

25(OH)D levels	Sunlight exposure*		*Use of sunscreen*		*Vitamin D food source*	
	3 or more times a week	Fewer than 3 times a week	Yes	No	Frequent	Infrequent
*Deficient*	2 (5.8)	51 (44.3)	16 (37.2)	40 (38)	5 (27.7)	48 (37.2)
*Insufficient*	13 (38.2)	52 (45.2)	14 (32.5)	47 (44.7)	6 (33.3)	57 (44.2)
*Normal*	19 (55.8)	12 (10.4)	13 (30.2)	18 (17.1)	7 (38.8)	24 (18.6)
Total	34 (100)	115 (100)	43 (100)	105 (100)	18 (100)	129 (100)

Values are expressed as number of patients (%); KT: kidney transplant; 25(OH)D: 25 hydroxyvitamin D;

* Chi-square test: *p*<0.0001.

**Table 4:** Biochemical parameters of patients undergoing KT according to 25(OH)D category.

	Deficient	Insufficient	Normal	*p*
	53	60	31	
Creatinine (mg/dL)	2.0 ± 0.9	1.7 ± 0.7	1.5 ± 0.5	0.013*
	1.7 (1.0 - 4.5)	1.5 (0.6 - 4.1)	1.3 (1.0 - 2.7)	
	52	60	31	
Albumin (g/dL)	4.4 ± 0.5	4.4 ± 0.3	4.4 ± 0.4	0.660
	4.4 (3.4 - 5.3)	4.4 (3.8 - 5.2)	4.5 (3.4 - 5.5)	
	51	59	31	
Ca (mg/dL)	8.9 ± 0.7	8.9 ± 0.5	8.9 ± 0.5	0.997
	9.0 (7.0 - 10.2)	8.9 (7.8 - 9.9)	8.8 (7.8 - 10.1)	
	55	61	31	
P (mg/dL)	3.0 ± 0.8	3.0 ± 0.8	2.7 ± 0.6	0.154
	2.9 (1.2 - 5.4)	2.9 (1.3 - 5.3)	2.7 (1.2 - 3.8)	
	55	61	31	
PTHi (pg/dL)	104.7 ± 104.6	72.5 ± 58.6	51.6 ± 37.0	0.011*
	68.6 (18.2 - 562.5)	53.2 (9.5 - 277.0)	44.1 (13.3 - 229.8)	
	53	60	31	
ALP (U/L)	82.8 ± 54.5	71.6 ± 30.3	65.8 ± 23.4	0.441
	69.6 (29.7 - 364.5)	64.8 (34.2 - 191.0)	65.6 (34.5 - 166.4)	
	52	60	31	
GOT (U/L)	27.9 ± 22.8	27.8 ± 11.7	23.5 ± 12.2	0.445
	21 (6 - 121)	25 (9 - 64)	19 (13 - 67)	
	53	60	31	
GPT (U/L)	14.2 ± 13.2	13.3 ± 8.5	13.2 ± 14.2	0.387
	10.0 (4 - 81)	10.5 (3 - 44)	9.0 (4 - 77)	
	51	58	31	
GGT (U/L)	67.5 ± 116.9	44.1 ± 50.1	35.6 ± 37.2	0.324
	31 (7 - 704)	28 (10 - 334)	22 (12 - 206)	

Values are expressed as number of patients, mean±SD and median (range). KT: kidney transplant; 25(OH)D: 25 hydroxyvitamin D; Ca: calcium; P: phosphorus; PTHi: intact parathyroid hormone; ALP: alkaline phosphatase; GOT: glutamic-oxaloacetic transaminase; GPT: glutamic-pyruvic transaminase; GGT: gamma-glutamyltransferase.

* Bonferroni’s post-hoc test: p<0.05, Normal vs. Deficient or Insufficient.

**Table 5:** Comparison of demographic and biochemical parameters between the control group and patients undergoing KT.

	Control group	Patients undergoing KT	*p*
Age	N=395	N=149	
18 to 30 years	68 (17)	29 (19)	0.078
31 to 50 years	210 (53)	71 (48)	
51 to 65 years	97 (25)	46 (31)	
More than 65 years	20 (5)	3 (2)	
Gender	N=398	N=149	
Female	333 (84)	84 (56)	<0.001
Male	65 (16)	65 (44)	
Race	N=385	N=149	
White	269 (68)	102 (68)	0.151
Non-white	116 (29)	47 (32)	
	N=348	N=149	
BMI (kg/m2)	25.9 ± 4.9	25.4 ± 5.3	0.118
	25.2 (16.8 - 56.3)	24.9 (16.4 - 44.0)	
	N=382	N=145	
Creatinine (mg/dL)	0.8 ± 0.1	1.8 ± 0.7	<0.001
	0.8 (0.4 - 1.2)	1.5 (0.6 - 4.5)	
	N=384	N=144	
Albumin (g/dL)	4.5 ± 0.3	4.4 ± 0.4	<0.001
	4.5 (3.3 - 5.4)	4.4 (3.4 - 5.5)	
	N=382	N=142	
Ca (mg/dL)	9.6 ± 0.5	8.9 ± 0.6	<0.001
	9.6 (6.7 - 10.8)	8.9 (7.0 - 10.2)	
	N=383	N=147	
P (mg/dL)	3.8 ± 0.7	3.0 ± 0.8	<0.001
	3.8 (1.8 - 7.6)	2.9 (1.2 - 5.4)	
	N=352	N=148	
PTHi (pg/dL)	64.9 ± 24.8	80.1 ± 78.3	<0.001
	60.5 (5.0 - 144.0)	53.9 (9.5 - 562.5)	
	N=398	N=149	
25(OH)D (ng/mL)	23.8 ± 10.4	21.8 ± 12.8	0.056
	21.6 (6.0 - 64.3)	20.7 (4.0 - 70.5)	
	N=381	N=145	
ALP (U/L)	70.9 ± 26.1	74.4 ± 40.1	0.306
	69.0 (24.0 - 369.0)	65.8 (29.7 - 364.5)	

Values are expressed as number of patients (%), mean±SD and median (range). KT: kidney transplant; BMI: body mass index; Ca: calcium; P: phosphorus; PTHi: intact parathyroid hormone; 25(OH)D: 25 hydroxyvitamin D; ALP: alkaline phosphatase.

**Table 6:** Patients undergoing KT and the control group according to 25(OH)D category.

25(OH)D levels	Control group	Patients undergoing KT	*p*
*Deficient*	90 (23)	56 (37)	0.001
*Insufficient*	209 (52)	62 (42)	
*Normal*	99 (25)	31 (21)	

Values are expressed as number of patients (%); KT: kidney transplant; 25(OH)D: 25 hydroxyvitamin D.

## References

[1] Holick MF (2007). Vitamin D deficiency. N Engl J Med.

[2] Unger MD, Cuppari L, Titan SM, Magalhães MC, Sassaki AL, dos Reis LM (2010). Vitamin D status in a sunny country: where has the sun gone. Clin Nutr.

[3] Binkley N, Novotny R, Krueger D, Kawahara T, Daida YG, Lensmeyer G (2007). Low vitamin D status despite abundant sun exposure. J Clin Endocrinol Metab.

[4] Çankaya E, Bilen Y, Keleş M, Uyanık A, Akbaş Ӎ, Güngör A (2015). Comparison of serum vitamin D Levels among patients with chronic kidney disease, patients in dialysis, and renal transplant patients. Transplant Proc.

[5] Pilz S, Iodice S, Zittermann A, Grant WB, Gandini S (2011). Vitamin D status and mortality risk in CKD: a meta-analysis of prospective studies. Am J Kidney Dis.

[6] Sadlier DM, Magee CC (2007). Prevalence of 25(OH) vitamin D (calcidiol) deficiency at time of renal transplantation: a prospective study. Clin Transplant.

[7] Stavroulopoulos A, Cassidy MJ, Porter CJ, Hosking DJ, Roe SD (2007). Vitamin D status in renal transplant recipients. Am J Transplant.

[8] Lomonte C, Antonelli M, Vernaglione L, Cazzato F, Casucci F, Chimienti D (2005). Are low plasma levels of 25-(OH)vitamin D a major risk factor for hyperparathyroidism independent of calcitriol in renal transplant patients. J Nephrol.

[9] Boudville NC, Hodsman AB (2006). Renal function and 25-hydroxyvitamin D concentrations predict parathyroid hormone levels in renal transplant patients. Nephrol Dial Transplant.

[10] Lim WH, Coates PS, Russ GR, Coates PT (2009). Hyperparathyroidism and vitamin D deficiency predispose to bone loss in renal transplant recipients. Transplantation.

[11] Eyal O, Aharon M, Safadi R, Elhalel MD (2013). Serum vitamin D levels in kidney transplant recipients: the importance of an immunosuppression regimen and sun exposure. Isr Med Assoc J.

[12] Euvrard S, Kanitakis J, Claudy A (2003). Skin cancers after organ transplantation. N Engl J Med.

[13] Obi Y, Hamano T, Ichimaru N, Tomida K, Matsui I, Fujii N (2014). Vitamin D deficiency predicts decline in kidney allograft function: a prospective cohort study. J Clin Endocrinol Metab.

[14] Levey AS, Bosch JP, Lewis JB, Greene T, Rogers N, Roth D (1999). A more accurate method to estimate glomerular filtration rate from serum creatinine: a new prediction equation. Modification of Diet in Renal Disease Study Group. Ann Intern Med.

[15] Ebbert K, Chow J, Krempien J, Matsuda-Abedini M, Dionne J (2015). Vitamin D insufficiency and deficiency in pediatric renal transplant recipients. Pediatr Transplant.

[16] Cofán F, Vela E, Clèries M, Catalan Renal Registry (2005). Obesity in renal transplantation: analysis of 2691 patients. Transplant Proc.

[17] Gore JL, Pham PT, Danovitch GM, Wilkinson AH, Rosenthal JT, Lipshutz GS (2006). Obesity and outcome following renal transplantation. Am J Transplant.

[18] Penny H, Frame S, Dickinson F, Garrett G, Young AR, Sarkany R (2012). Determinants of vitamin D status in long-term renal transplant patients. Clin Transplant.

[19] Palmer SC, Mc Gregor DO, Strippoli GF (2007). Interventions for preventing bone disease in kidney transplant recipients. Cochrane Database Syst Rev.

[20] Giannini S, Sella S, Silva Netto F, Cattelan C, Dalle Carbonare L, Lazzarin R (2010). Persistent secondary hyperparathyroidism and vertebral fractures in kidney transplantantion: role of calcium-sensing receptor polymorphisms and vitamin D deficiency. J Bone Min Res.

[21] Bischoff-Ferrari HA, Willett WC, Wong JB, Stuck AE, Staehelin HB, Orav EJ (2009). Prevention of nonvertebral fractures with oral vitamin D and dose dependency: a meta-analysis of randomized controlled trials. Arch Intern Med.

[22] Hesketh CC, Knoll GA, Molnar AO, Tsampalieros A, Zimmerman DL (2014). Vitamin D and kidney transplant outcomes: a protocol for a systematic review and meta-analysis. Syst Rev.

[23] Attard NR, Karran P (2012). UVA photosensitization of thiopurines and skin cancer in organ transplant recipients. Photochem Photobiol Sci.

[24] Moloney FJ, Comber H, O'Lorcain P, O'Kelly P, Conlon PJ, Murphy GM (2006). A population-based study of skin cancer incidence and prevalence in renal transplant recipients. Br J Dermatol.

[25] Reichrath J (2006). The challenge resulting from positive and negative effects of sunlight: how much solar UV exposure is appropriate to balance between risks of vitamin D deficiency and skin cancer. Prog Biophys Mol Biol.

[26] Moan J, Porojnicu AC, Dahlback A, Setlow BR (2008). Addressing the health benefits and risks, involving vitamin D or skin cancer, of increased sun exposure. Proc Natl Acad Sci U S A.

[27] Ferreira FR, Ogawa MM, Nascimento LF, Tomimori J (2014). Epidemiological profile of nonmelanoma skin cancer in renal transplant recipients: experience of a referral center. An Bras Dermatol.

[28] Filipov JJ, Zlatkov BK, Dimitrov EP, Svinarov D (2015). Relationship between vitamin D status and immunosuppressive therapy in kidney transplant recipients. Biotechnol Biotechnol Equip.

[29] Eyal O, Aharon M, Safadi R, Elhalel MD (2013). Serum vitamin D Levels in kidney transplant recipients: the importance of an immunosuppression regimen and Sun exposure. Isr Med Assoc J.

[30] Kamen DL, Tangpricha V (2010). Vitamin D and molecular actions on the immune system: modulation of innate and autoimmunity. J Mol Med.

[31] Sezer S, Yavuz D, Canoz MB, Ozdemir FN, Haberal M (2009). Vitamin D status, bone mineral density, and inflammation in kidney transplantation patients. Transplant Proc.

[32] Bienaimé F, Girard D, Anglicheau D, Canaud G, Souberbielle JC, Kreis H (2013). Vitamin D status and outcomes after renal transplantation. J Am Soc Nephrol.

